# Fecal Volatile Organic Ccompound Profiles from White-Tailed Deer (*Odocoileus virginianus*) as Indicators of *Mycobacterium bovis* Exposure or *Mycobacterium bovis* Bacille Calmette-Guerin (BCG) Vaccination

**DOI:** 10.1371/journal.pone.0129740

**Published:** 2015-06-10

**Authors:** Randal S. Stahl, Christine K. Ellis, Pauline Nol, W. Ray Waters, Mitchell Palmer, Kurt C. VerCauteren

**Affiliations:** 1 United States Department of Agriculture (USDA)-Animal and Plant Health Inspection Service (APHIS)-Wildlife Services, National Wildlife Research Center, Fort Collins, Colorado, United States of America; 2 Wildlife Livestock Disease Investigations Team, USDA-APHIS-Veterinary Services-Science, Technology, and Analysis Services, National Veterinary Services Laboratory, Fort Collins, Colorado, United States of America; 3 Infectious Bacterial Diseases Research Unit, USDA-Agricultural Research Service, National Animal Disease Center, Ames, Iowa, United States of America; University of Minnesota, UNITED STATES

## Abstract

White-tailed deer (*Odocoileus virginianus*) serve as a reservoir for bovine tuberculosis, caused by *Mycobacterium bovis*, and can be a source of infection in cattle. Vaccination with *M*. *bovis* Bacille Calmette Guerin (BCG) is being considered for management of bovine tuberculosis in deer. Presently, no method exists to non-invasively monitor the presence of bovine tuberculosis in deer. In this study, volatile organic compound profiles of BCG-vaccinated and non-vaccinated deer, before and after experimental challenge with *M*. *bovis* strain 95–1315, were generated using solid phase microextraction fiber head-space sampling over suspended fecal pellets with analysis by gas chromatography/mass spectrometry. Chromatograms were processed using XCMS Online to characterize ion variation among treatment groups. The principal component scores resulting from significant (α = 0.05) ion responses were used to build linear discriminant analysis models. The sensitivity and specificity of these models were used to evaluate the feasibility of using this analytical approach to distinguish within group comparisons between pre- and post-*M*. *bovis* challenge: non-vaccinated male or female deer, BCG-vaccinated male deer, and the mixed gender non-vaccinated deer data. Seventeen compounds were identified in this analysis. The peak areas for these compounds were used to build a linear discriminant classification model based on principal component analysis scores to evaluate the feasibility of discriminating between fecal samples from *M*. *bovis* challenged deer, irrespective of vaccination status. The model best representing the data had a sensitivity of 78.6% and a specificity of 91.4%. The fecal head-space sampling approach presented in this pilot study provides a non-invasive method to discriminate between *M*. *bovis* challenged deer and BCG-vaccinated deer. Additionally, the technique may prove invaluable for BCG efficacy studies with free-ranging deer as well as for use as a non-invasive monitoring system for the detection of tuberculosis in captive deer and other livestock.

## Introduction

Bovine tuberculosis (bTB), caused by *Mycobacterium bovis*, is a disease of importance to public health, domestic agriculture, and international trade [1, 2]. Implementation of disease surveillance and eradication programs in the United States (US) has dramatically reduced the prevalence of bTB in domestic livestock herds [3]; however, import of infected animals from Mexico, infrequent inter-herd transmission (including transmission from captive cervids to cattle), and the endemic presence of bTB in free-ranging populations of white-tailed deer (*Odocoileus virginianus;* WTD) in Michigan, USA and feral swine (*Sus scrofa*) on Molokai Island, Hawaii, USA have been major obstacles to achieving disease-free status [4–7]. According to the World Health Organization (WHO), approximately 8.8 million incident cases of human tuberculosis occurred globally in 2010 [8]. *Mycobacterium tuberculosis* was responsible for the majority of those cases; however, an unknown proportion of cases were likely attributable to *M*. *bovis* [9, 10]. Eradication programs [11] and milk pasteurization have decreased the incidence of bTB in developed countries; however, in some developing countries, disease prevalence in cattle may exceed 10% [12, 13].

Bovine tuberculosis is endemic at low prevalence in the WTD population in northeastern Michigan, which serves as a reservoir for transmission to cattle [14, 15]. Surveillance data identified core outbreak areas with a prevalence rate of approximately 2%, with focal areas within the core area having higher prevalence (> 3.5%)[16]. Primary surveillance and control strategies for WTD have historically relied on reducing WTD densities through hunting and by restricting baiting and supplemental feeding. Oral vaccination with *M*. *bovis* Bacille Calmette-Guerin (BCG) has been shown to be effective in protecting WTD from disease and is being considered as a management tool in addition to the existing tools already in place [17–19].

Standard procedures for monitoring captive cervids for bTB are based on the administration of a single cervical tuberculin test (SCT) followed by a comparative cervical tuberculin test (CCT) [20] and more recently, the Dual Path Platform VetTB Assay (DPP; Chembio Diagnostic Systems, Inc., Medford, NY, USA) which has been approved for use as both primary and secondary tests [21]. These testing strategies require one or more animal handling events and DPP-based approaches may falsely identify BCG-vaccinated animals as *M*. *bovis*-infected [18, 22]. The United States Department of Agriculture (USDA) performed a comprehensive evaluation in cervids of the SCT and CCT used in series and reported that the sensitivity and specificity were 87.1 and 90.4% respectively [23]. Palmer et al. [24] demonstrated the sensitivity and specificity of the CCT alone to be 97% and 81% respectively in 169 known infected and non-infected WTD. The estimated sensitivity and specificity of DPP in WTD was 65% and 98%, respectively [25].

Detection of disease-specific volatile organic compounds (VOCs) present in breath or feces could allow for testing of captive WTD with minimal handling and has great potential for use in remote disease surveillance of wildlife. In this pilot study, we assessed the feasibility of discriminating between non-vaccinated and BCG-vaccinated WTD prior to and five months post-experimental challenge with *M*. *bovis* based on gas chromatography/mass spectrometry (GC/MS) analysis of fecal VOC profiles.

## Materials and Methods

### Ethics Statement

Strict biosafety level 3 (BL-3) safety protocols were followed during all challenge and animal handling procedures to protect personnel from exposure to *M*. *bovis*. All animal work was reviewed and approved by the Institutional Biosafety and Animal Care and Use Committees (IACUC) of the USDA, Agricultural Research Service (ARS), National Animal Disease Center (NADC), Ames, Iowa, USA and the USDA, Animal and Plant Health Inspection Service (APHIS), National Wildlife Research Center (NWRC), Fort Collins, Colorado, USA prior to initiation of studies.

### Animals and *Mycobacterium bovis* challenge

Twelve to eighteen month-old castrated male and intact female WTD were obtained from a bTB free, captive-herd at NADC for use in a BCG vaccine efficacy trial involving experimental challenge with *M*. *bovis*. Deer were randomized across two treatment groups: a non-vaccinated control group (n = 16), and a treatment group vaccinated with BCG Danish (n = 17). After an initial observation period, animals in the BCG-vaccinated group were restrained in a drop floor chute and vaccinated by depositing a liquid suspension of BCG vaccine in the posterior oral cavity as described in [17].

Three months post-vaccination, all treatment groups were transferred to segregated rooms in the BSL-3 animal facility at NADC. Each treatment group was housed according to IACUC guidelines in separate biocontainment rooms with no exchange of air, feed, or water occurring between rooms. All animals were housed under the same environmental conditions and fed the same diet. After a two week acclimation period, all WTD were anesthetized using xylazine (2 mg/kg; Mobay Corporation, Shawnee, KS, USA) and ketamine (6 mg/kg; Fort Dodge Laboratories, Fort Dodge, IA, USA) administered by intramuscular (IM) injection. *Mycobacterium bovis* challenge was administered by intratonsilar inoculation into each palatine tonsillar crypt [26]. Challenge inoculum consisted of 150 colony-forming units (CFU) of *M*. *bovis* strain 95–1315 (USDA, APHIS designation) prepared using standard procedures in Middlebrook 7H9 liquid media (Becton Dickinson, Franklin Lakes, NJ, USA) and delivered in a final challenge dose of 300 CFU per WTD as described in Palmer et al [26]. After challenge, the effects of xylazine were reversed using tolazoline (4 mg/kg; Lloyd Laboratories, Shenandoah, IA, USA) administered IM.

Three months post-challenge, five WTD from both the non-vaccinated control and BCG vaccinated treatment groups were euthanized and examined. Samples from these animals were not included in this study. Five months post-challenge, all remaining WTD were euthanized and examined. All animals were euthanized by intravenous administration of sodium pentobarbital while restrained in the drop chute.

### Samples Collected

Fecal samples were opportunistically collected *per rectum* from WTD across the vaccination treatment groups for GC/MS analysis according to the schedule presented in Table 1. Samples were placed in 50 ml conical centrifuge tubes and stored at -80°C prior to shipment to NWRC. Samples were shipped on dry ice, and then stored at -80°C until analysis. Tissue samples collected from all WTD at necropsy for pathology scoring, histopathology, and isolation and identification of *M*. *bovis* as previously described [26], included lung; liver; palatine tonsil; and mandibular, parotid, medial retropharyngeal, tracheobronchial, mediastinal, hepatic, mesenteric, and superficial cervical lymph nodes.

**Table 1:** Study design for the number of samples collected for GC/MS analysis by treatment group.

Treatment	Sex	Sampled prior to the *M*. *bovis* challenge	Sampled 5 months post-challenge
non-vaccinated	Male	n = 4	n = 4
	Female	n = 3	n = 3
BCG-vaccinated	Male	n = 4*	n = 4
	Female	-	-

*-Occurred three months post-BCG-vaccination.

### Sample Preparation for GC/MS Analysis

All fecal sample processing was performed in a Biosafety Level II laboratory in a Biosafety Class II cabinet by a trained researcher wearing appropriate personal protective equipment (PPE). Fecal slurries were prepared by suspending one fecal pellet (~ 0.50 g) in 5.0 mL phosphate buffered saline (PBS; pH = 7.0) in a 15.0 ml vial with a phenolic screw-top and polytetrafluoroethylene (PTFE) septum containing a micro stir bar to ensure adequate mixing. Vials were vortexed to create a fecal slurry, and then placed on a heated stir plate at 60°C for 30.0 minutes. During this interval, headspace VOC sampling was performed using a 24Ga 50/30 μm divinylbenzene/carboxen/ polydimethylsiloxane (DVB/CAR/PDMS) solid phase microextraction (SPME) fiber (Supelco, St. Louis, MO, USA). The SPME fiber was preconditioned at 270°C for 1 h prior to collecting a sample.

### Sample Analysis by GC/MS

Analysis was performed using an Agilent 6890 GC coupled with an Agilent 5973 MS (Agilent Technologies, Santa Clara, CA, USA). The SPME fiber was manually inserted into the GC inlet port and the analytes were desorbed from the fiber for one minute at 240°C in splitless mode, at an inlet pressure of 103.4 kPa (15 psi). The carrier gas was helium delivered with an average velocity of 51 cm/s. The column used was a DB-5ms (J&W Scientific, Agilent Technologies, Santa Clara, CA, USA) 30 m x 250 μm column with a film thickness of 0.25 μm. Analytes were eluted from this column using a thermal gradient starting at 30°C and ramping at a rate of 5°C/min to a final temperature of 250°C. The total GC run time was 47.0 min. The temperature of the transfer line was 280°C. The MS was operated in positive ion mode, performing a total ion scan ranging from 50 to 550 m/z with a threshold of 150 m/z at a scan rate of 20 Hz. The MS source was operated at 230°C with the quad set to 150°C. Data were generated as raw Agilent.dat files.

Method performance was assessed by determining limits of detection (LOD) and linearity for three compounds observed in the chromatograms: 6-methyl-5-hepten-2-one, indole, and 1-octadecanol. Standard stock solutions were prepared in ethanol and spiked into PBS for a final volume of 5.0 mL. The diluted concentration ranges evaluated for linearity were 0.01–0.2 μg/mL for 6-methyl-5-hepten-2-one; 0.06–0.6 μg/mL for indole, and 0.9–9.0 μg/mL for 1-octadecanol. The lowest standard concentration used in the linear range determination for each of the compounds was used to estimate the LOD, calculated as a concentration that would produce a peak height 3 times the average baseline noise. Head space samples were collected from the spiked PBS using the procedure described above for the fecal samples. Three replicates were determined at each concentration over three days to assess inter- and intra-day variations in method performance. Inter-day results for the LOD were compared for significant differences using a one-way analysis of variance using”R” (http://www.r-project.org/).

### Data Analysis

Chromatograms were analyzed using XCMS Online (www.xcmsonline.scripps.edu) to identify VOCs present in the chromatograms that differed across treatment groups in observed peak ion abundances. Four pairwise within group comparisons were evaluated utilizing this method. The first comparison evaluated the differences in the chromatograms for fecal samples collected from non-vaccinated male WTD prior to and five months post- *M*. *bovis* challenge. The second comparison was between male BCG-vaccinated WTD three months post-vaccination prior to challenge and five months post-challenge. The third comparison was between the three non-vaccinated female WTD sampled pre-and five months post-challenge. The fourth comparison, to assess the effect of a mixed gender population, was performed using a population comprised of both the male and female non-vaccinated WTD sampled pre-challenge and five months post-challenge.

The ions identified as significantly different in each of the four within-group comparisons, evaluated pre- and post-challenge, were used in principle components analysis (PCA) and linear discriminant analysis (LDA) classification models using the “chemometrics” statistical package in “R” [27]. Individual ion intensities were median centered and scaled to a variance of 1.0 using the median absolute deviation. Data were evaluated for the presence of outliers, which were identified as exceeding the regular observations by the 97.5% quantile of a standard normal distribution of score distance and the orthogonal distance from the PCA space for removal from subsequent analyses with LDA classification models using 2 PCA scores being developed for each of the four comparisons.

The LDA classification models were written as two class models; classifying a sample as belonging to one of the two classes in each of the four comparisons. A training dataset was constructed by randomly subsampling five of the data sets, and a classification dataset was constructed from the remaining three data sets. The LDA classification was performed for 100 iterations and the resulting predicted classification of each test animal in a given iteration was compared to the actual treatment group assignment. Misclassification rates for each of the models were determined to ascertain the reliability of using a subset of ions measured from fecal samples as means of discriminating across the treatments.

We compared the ability of our LDA classification models to correctly identify treatment classes by calculating sensitivity (Sn) and specificity (Sp) using the PCA scores generated from the XCMS Online analysis. For each of the comparisons the numbers of true positive and true negative samples classified across 100 iterations of the classification simulation were summed. Samples that were misclassified as falsely positive (negative sample incorrectly classified as positive; non-vaccinated sample incorrectly classified as vaccinated) or falsely negative (positive sample incorrectly classified as negative; vaccinated sample incorrectly classified as non-vaccinated) were then summed. Sensitivity was calculated as the total number of true positives divided by the sum of the true positives plus false negatives. Specificity was calculated as the sum of all true negative samples divided by sum of the true negative plus false positive samples [28]. These values are reported as percentages.

To make comparisons between vaccination treatment groups, peaks identified as being significantly different (1.5 minimum fold increase, α < 0.05) based on ion intensity in the XCMS Online analysis were identified using the National Institute of Science and Technology (NIST) W8N08 database (www.nist.gov). Peak areas were determined for these compounds from the total ion chromatogram (TIC) for the sample using Chemstation and were incorporated into a new classification analysis to assess the relevance of these compounds as potential markers indicative of vaccination or disease status of an individual WTD. The approach was to build a classification model using PCA scores derived from a PCA analysis of the peak areas in either a two or three class LDA model.

The two class model was developed to predict vaccination status for the samples collected three months post vaccination and immediately prior to challenge. The peak areas were for analytes identified in samples from non-vaccinated male and female WTD and the vaccinated male WTD, with the data from both sexes of the non-vaccinated WTD combined in a single class.

In the three-class model, two of the classes were for fecal samples originating from the non-vaccinated WTD, the first being samples collected prior to *M*. *bovis* challenge and the second being samples collected five months post-challenge. The third class consisted of samples collected from BCG-vaccinated male WTD irrespective of challenge. Models were fit using two to five PCA scores. Data from samples identified as significant outliers orthogonal to the PCA space were removed from the model.

The LDA classification predictions for both the two and three class models were assessed using a data subset comprised of peak areas for three randomly selected fecal samples. The remaining fecal sample data were used to train the model over 100 iterations with the resulting predicted classification of each test animal in a given iteration of the model compared to the actual treatment group assignment. The LDA model misclassification rates for each of the three classes were assessed and sensitivity and specificity for the model were calculated.

## Results

### Diagnostic Samples

All the deer sampled for VOC analysis were challenged with *M*. *bovis*. Results of semi-quantitative scoring of gross lesions present in the lungs and lymph nodes, histopathology, and isolation and identification of mycobacterial isolates are reported elsewhere [26]. Briefly, no gross lesions were observed in 15/17 (88.2%) of the BCG-vaccinated deer. By comparison, no gross lesions were observed in 5/16 (31%) of the non-vaccinated group. Lesions, when present in BCG-vaccinated animals, were found only in the medial retropharyngeal lymph nodes; whereas, lesions were noted in the tracheobronchial, medial retropharyngeal, and mediastinal lymph nodes, and lungs of non-vaccinated deer. Microscopic lesions compatible with tuberculous granulomas were identified in 4/17 (24%) and 11/16 (69%) of BCG-vaccinated and non-vaccinated deer, respectively. *Mycobacterium bovis* was isolated from 5/17 (29%) BCG- vaccinated deer, and 9/16 (56%) non-vaccinated deer. For proof of concept, in this study, the four male BCG-vaccinates included in this analysis had no gross or microscopic lesions compatible with *M*. *bovis* experimental challenge, nor was *M*. *bovis* isolated from tissues. All four of the male non-vaccinates and the three female non-vaccinates sampled for analysis in this study developed detectible lesions and *M*. *bovis* was isolated from tissues.

### Cloud Plots

**Fig 1:**
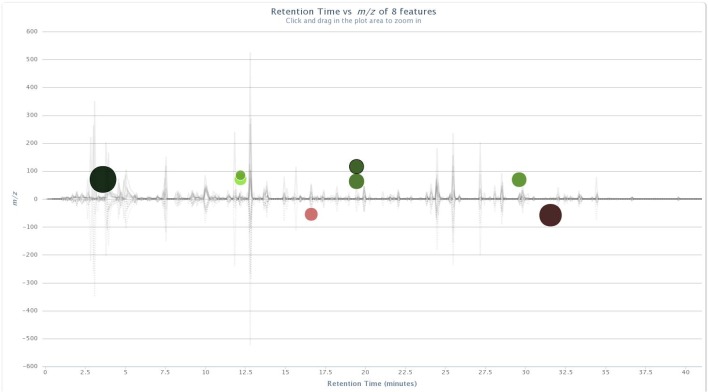
Cloud plot of aligned GC/MS chromatograms generated with XCMS Online for non-vaccinated male WTD pre-challenge vs. five months post-*M*. *bovis* strain 95–1315 challenge. Pre-challenge fecal sample chromatograms are depicted below the X-axis. Post- challenge chromatograms are positioned above. Up-regulated features of statistical significance are identified with green-colored circles located at the top of the plot, and down-regulated features are identified by red-colored circles located at the bottom of the plot. The color intensity of each circle represents the statistical significance of the feature difference, with brighter circles having lower p-values. The diameter of each circle represents a log-fold increase or decrease in abundance (i.e., larger circles correspond to peaks with greater fold differences). doi: http://dx.doi.org/10.6084/m9.figshare.141831.

**Fig 2:**
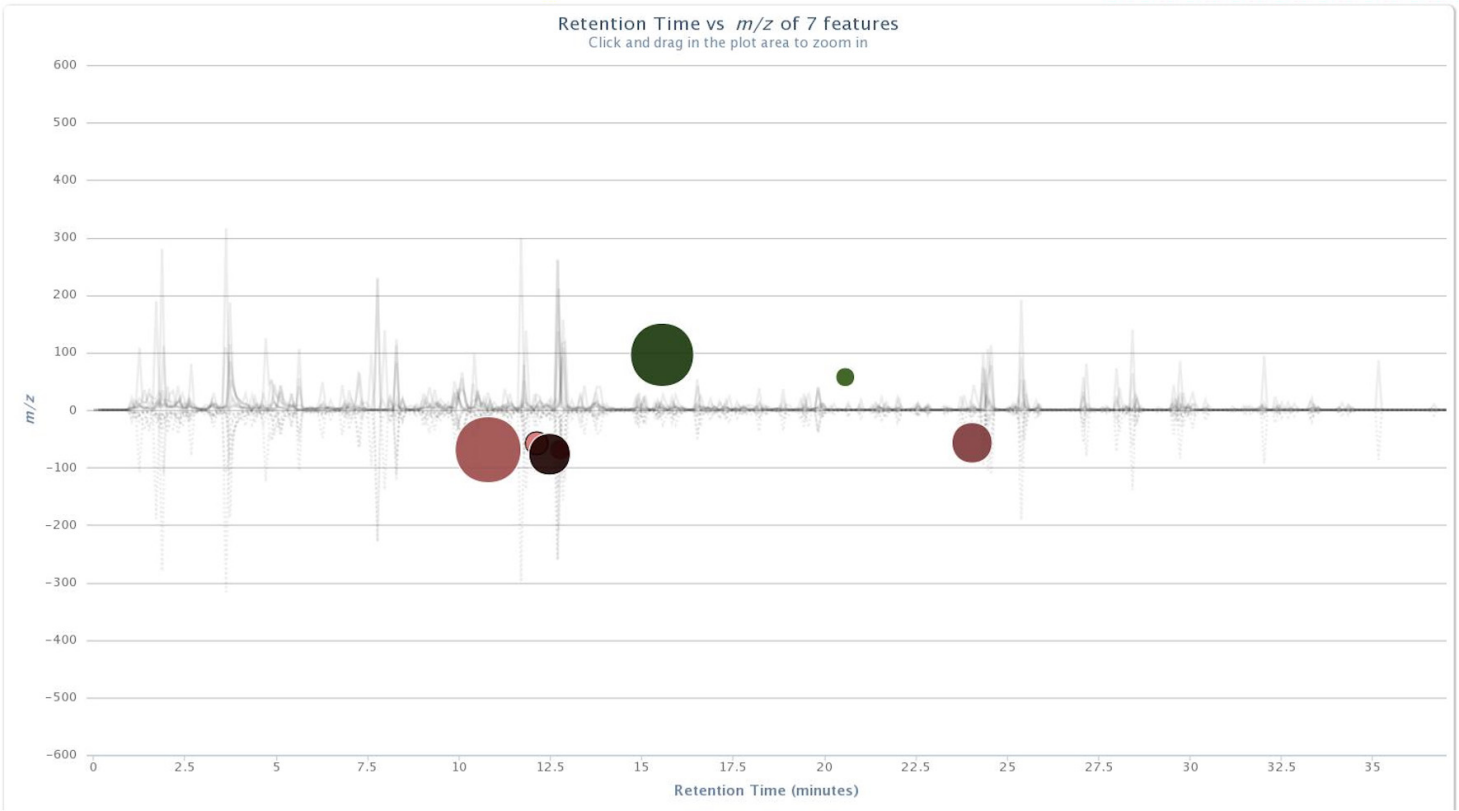
Cloud plots of aligned GC/MS chromatograms generated with XCMS Online for vaccinated male WTD pre-challenge vs. five months post-*M*. *bovis* strain 95–1315 challenge. doi: http://dx.doi.org/10.6084/m9.figshare.1418313.

**Fig 3:**
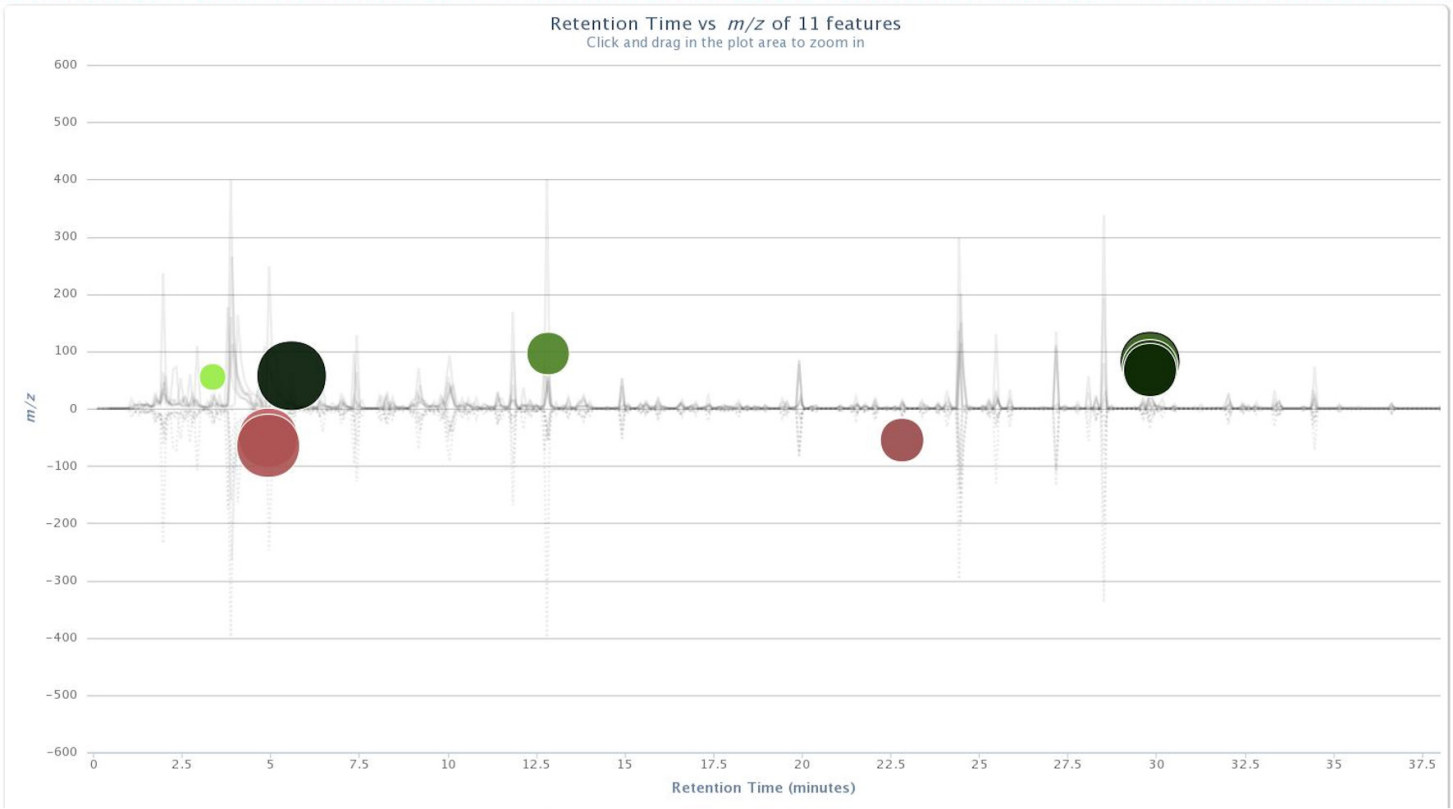
Cloud plots of aligned GC/MS chromatograms generated with XCMS Online for non-vaccinated female WTD pre-challenge vs. five months post-*M*. *bovis* strain 95–1315 challenge. doi: http://dx.doi.org/10.6084/m9.figshare.1418312.

**Fig 4:**
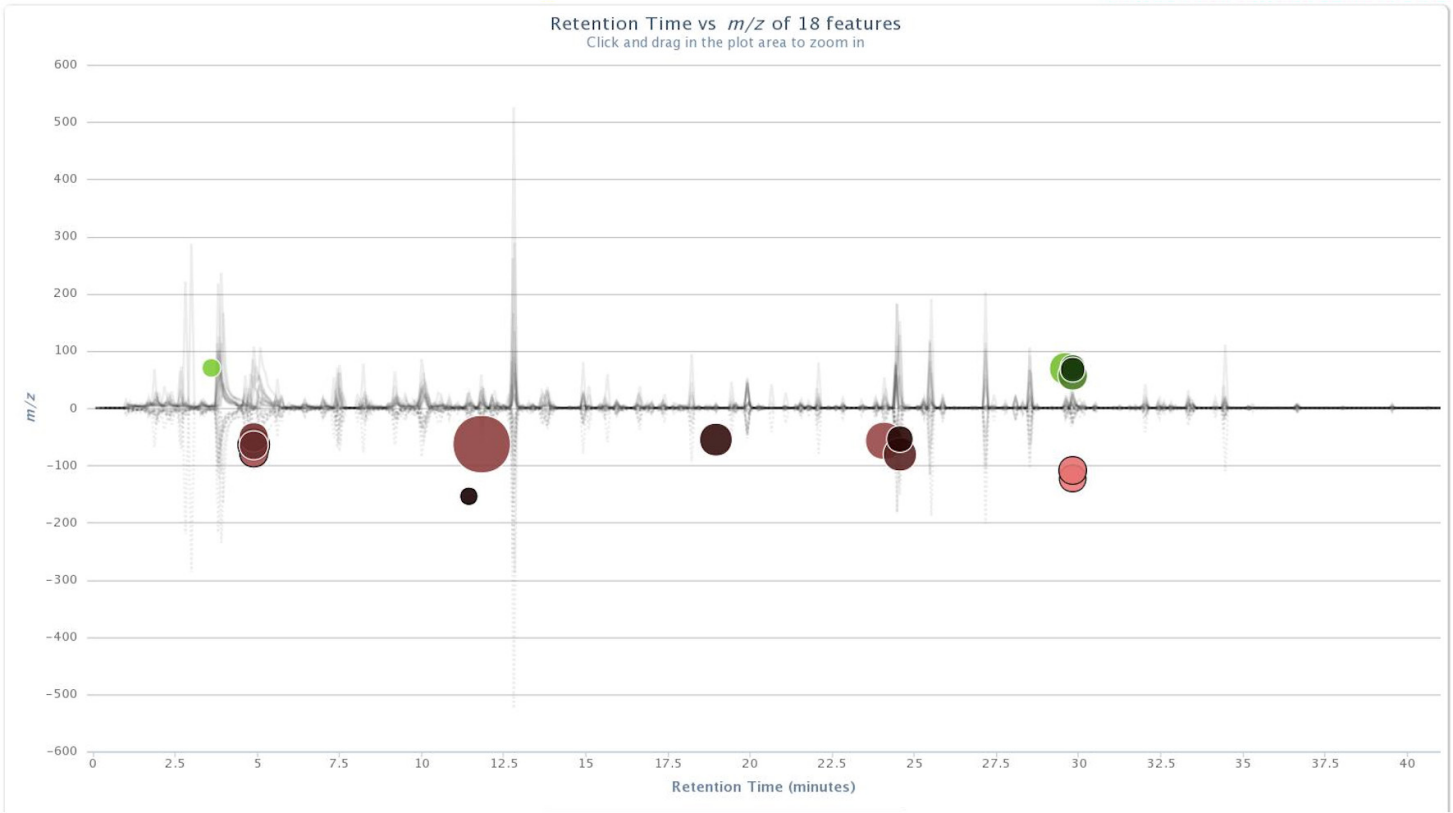
Cloud plots of aligned GC/MS chromatograms generated with XCMS Online for combined non-vaccinated male (n = 4) and female (n = 3) data pre-challenge vs. 5 months post-*M*. *bovis* strain 95–1315 challenge. doi: http://dx.doi.org/10.6084/m9.figshare.1418311.

Significant ion intensity differences across the treatment pair-wise comparisons are plotted as cloud plots in XCMS Online. Figs 1 and 2 depict the overlaid chromatograms for the within-treatment group comparisons of non-vaccinated and BCG-vaccinated male WTD pre-challenge and five months post-challenge. Fig 3 depicts the within-treatment group pre- and post-challenge comparisons for the non-vaccinated female WTD, while Fig 4 depicts the combined non-vaccinated mixed gender WTD comparison. For the non-vaccinated male comparison eight ions were significantly different across the comparison (p<0.05, fold intensity >1.5 change) out of 723 ions identified as different between the two sampling intervals. For the BCG-vaccinated male comparison seven ions were identified as significantly different out of 756 total ions identified across the comparison. For the non-vaccinated female comparison 11 ions were identified as significant out of 434 ions, and for the mixed gender analysis 18 ions were identified as significant out of 471 ions. The ions identified by these comparisons were then used to parameterize the respective classification models for each group comparison following PCA transformation.

The cloud plots (Figs 1–4) visually convey the fold change in ion intensity as well as the level of significance. The fold increase in the significantly different ion intensities identified in the samples collected from the non-vaccinated male WTD ranged from a 2.6 fold increase at 12.1 min to a 13.6 fold increase at 3.6 min (Fig 1). Fold increases of 2.8 at 20.5 min up to a 31.6 fold increase at 15.5 min were observed in ion intensity for the comparison of the BCG-vaccinated male WTD (Fig 2). The fold increases in ion intensity observed in the non-vaccinated female WTD ranged from 4.3 at 29.8 min to 57.7 at 5.6 min. The fold change in significantly different ion intensities for the mixed gender non-vaccinated WTD data ranged from 2.2 at 11.4 min to 15.7 at 11.8 min.

### Compound Identification

The ions that were significantly different across the treatment groups identified in the XCMS Online cloud plots (Figs 1–4) were tentatively identified using Agilent ChemStation software and the NIST W8N08 mass spectral library using the corresponding retention times of the ions, and are listed in Table 2. Ions identified as significantly different in intensity by treatment group in Figs 1–4 that could not be identified due to poor library search match results are not included.

**Table 2:** Statistically significant trends identified for compounds identified by VOC head space analysis for samples collected pre-challenge and 5 months post-challenge across all within treatment group comparisons.

		Vaccination Status			
		Non-vaccinated	Vaccinated	Non-vaccinated	Non-vaccinated
		Sex			
Compound	Retention Time (min)	Male (n = 4)	Male (n = 4)	Female (n = 3)	Mixed (n = 7)
Methylbenzene	3.9	Increased		Increased	Increased
Hexanal	4.5			Decreased	Decreased
2-Methyl pyridine	4.9			Decreased	Decreased
2,4-Dimethyl pyridine	5.5			Increased	
2-(1,1-Dimethoxy)-ethanol	10.8		Decreased		
2-Ethyl-1-hexanol	11.4				Decreased
Benzene acetaldehyde	11.8				Decreased
3,7-Dimethyl-6-octenyl-(2E)-2-butanoate	12.1	Increased	Decreased		
Acetophenone	12.5		Decreased		
4-Methyl-phenol	12.8		Decreased	Increased	
2-Decanone	15.6		Increased		
(-)-Beta-Fenchol	16.7	Decreased			
1-Decanol	18.9				Decreased
Indole	19.4	Increased			
3-(1,1-dimethylethyl)-4-methoxy-phenol	24.0		Decreased		Decreased
1-Octadecanol	29.6	Increased		Increased	Increased
2-Dodecanone	31.5	Decreased			

Two of the compounds used to evaluate method sensitivity and instrument response to concentration, indole and 1-octadecanol, appear in Table 2 and were observed to elute at 18.9 and 29.6 min, respectively. 6-Methyl-5-hepten-2-one appeared at a retention time of 9.4 min but was not impacted by vaccination status or *M*. *bovis* challenge. The mean LOD’s estimated for the compounds were 0.00023μg/mL for 6-methyl-5-hepten-2-one, 0.0028 μg/mL for indole, and 0.15 μg/mL for 1-octadecanol, and the inter day LOD’s were not significantly different across three replicates.

### Classification Models

**Fig 5:**
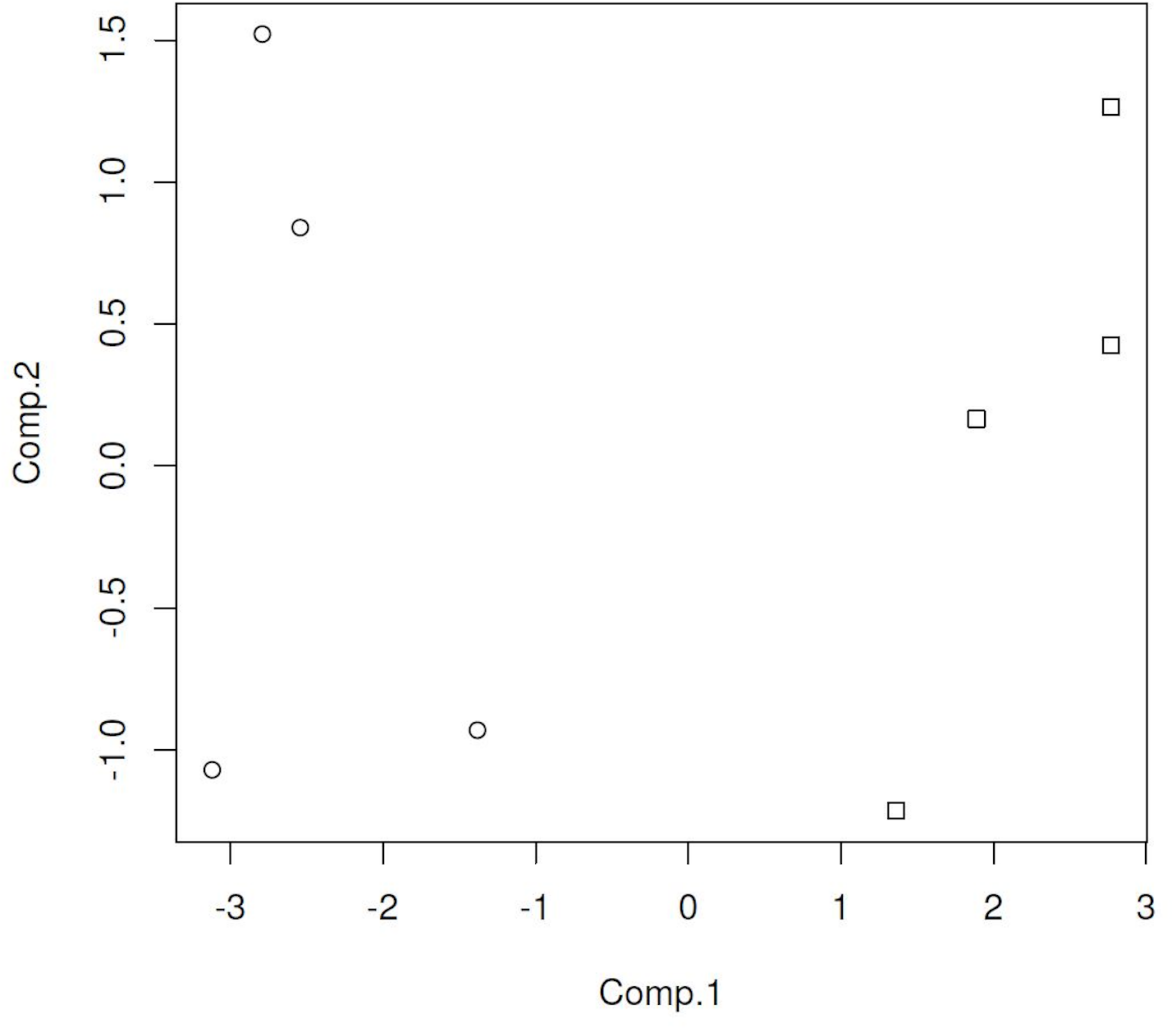
PCA score plot for non-vaccinated WTD fecal sample cluster analysis. On the X-axis are the 1^st^ component scores, the on the Y-axis are the 2nd component scores. Squares represent pre-challenge samples, circles represent post-challenge samples at 5 months. doi: http://dx.doi.org/10.6084/m9.figshare.141831.

**Fig 6:**
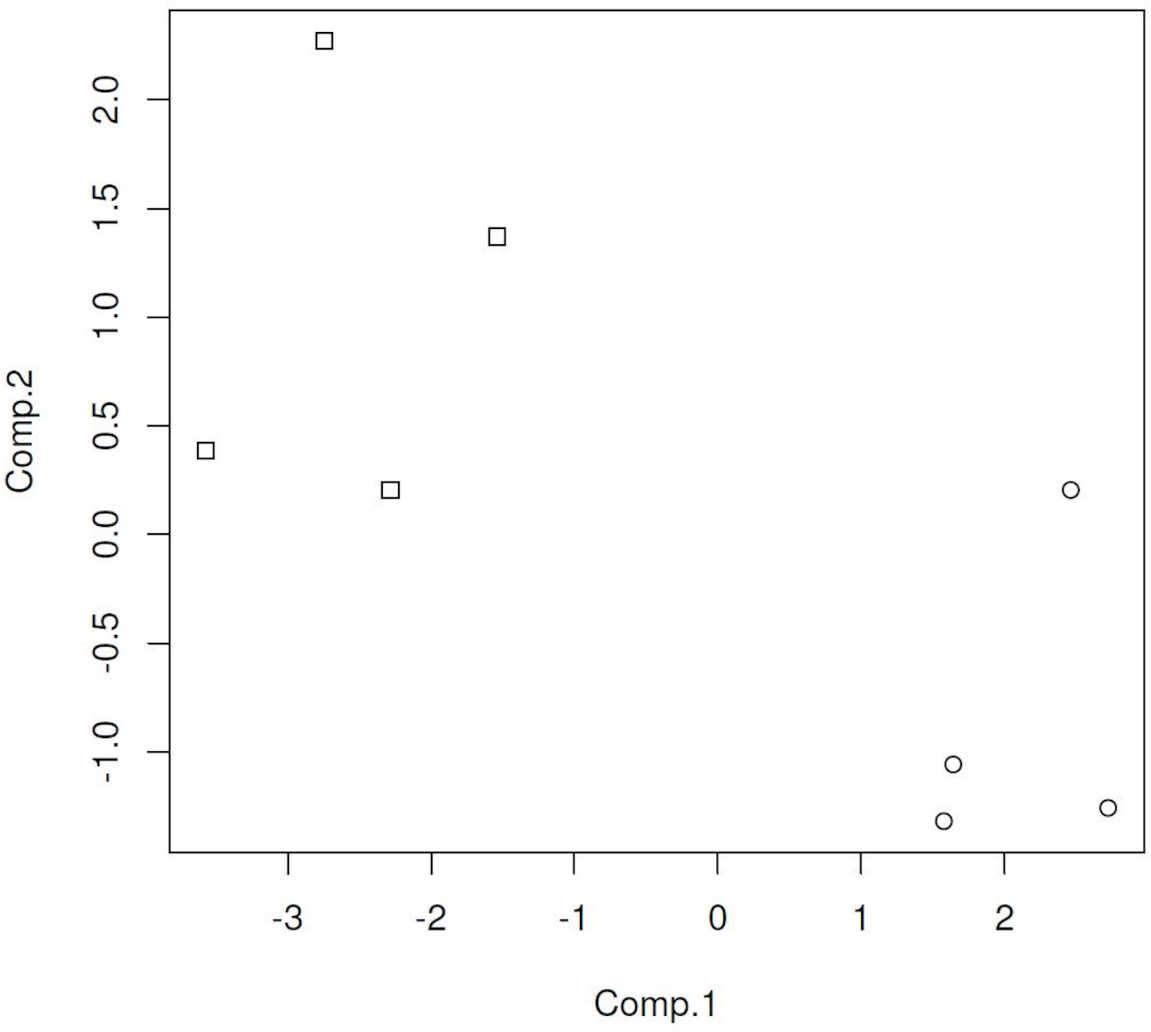
PCA score plot for vaccinated deer fecal sample cluster analysis. Squares represent the pre challenge samples, circles the post challenge samples at 5 months. doi: http://dx.doi.org/10.6084/m9.figshare.1418313.

**Fig 7:**
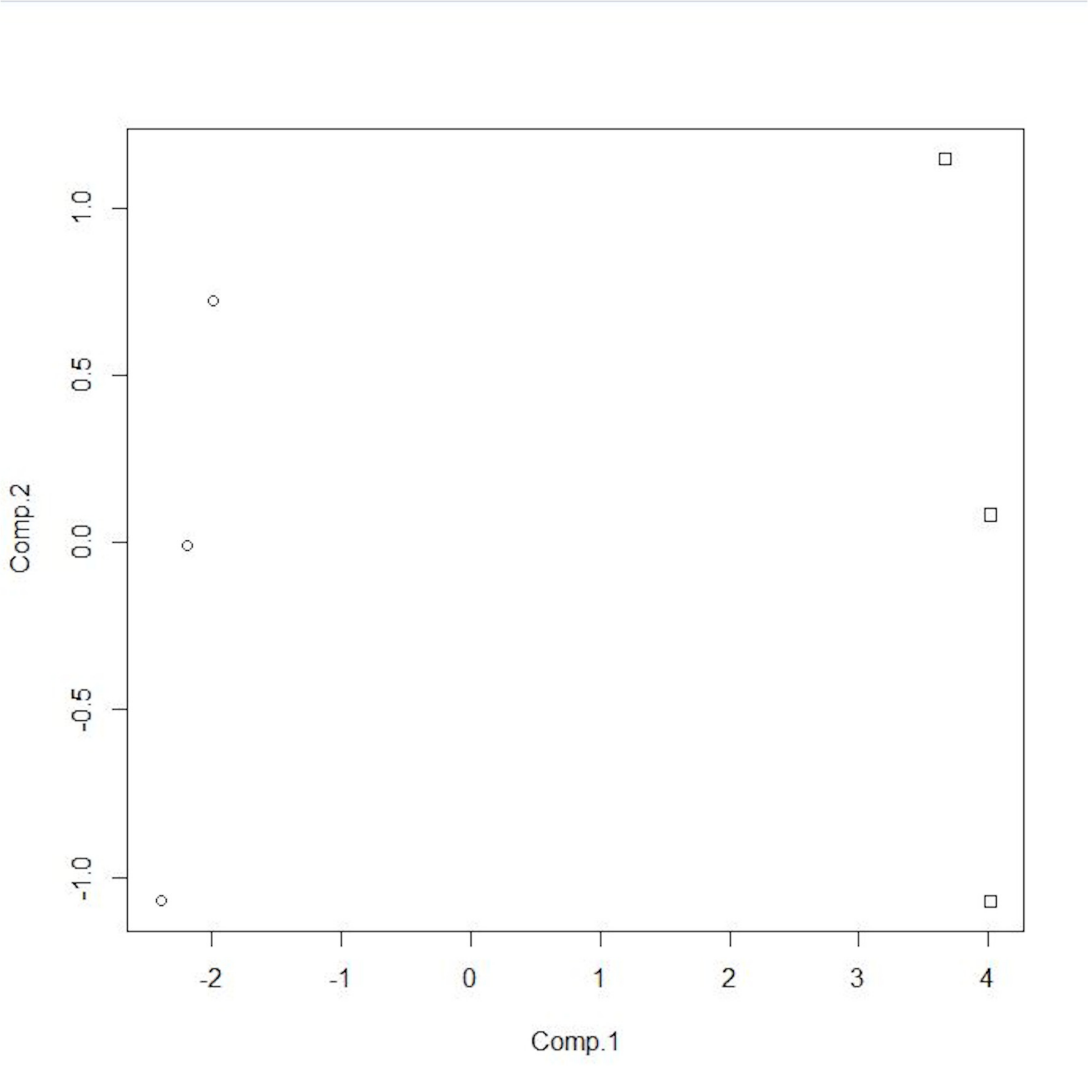
PCA score plot for non-vaccinated female WTD. Squares represent the pre-challenge samples, circles the post-challenge samples at 5 months. doi: http://dx.doi.org/10.6084/m9.figshare.1418312.

**Fig 8:**
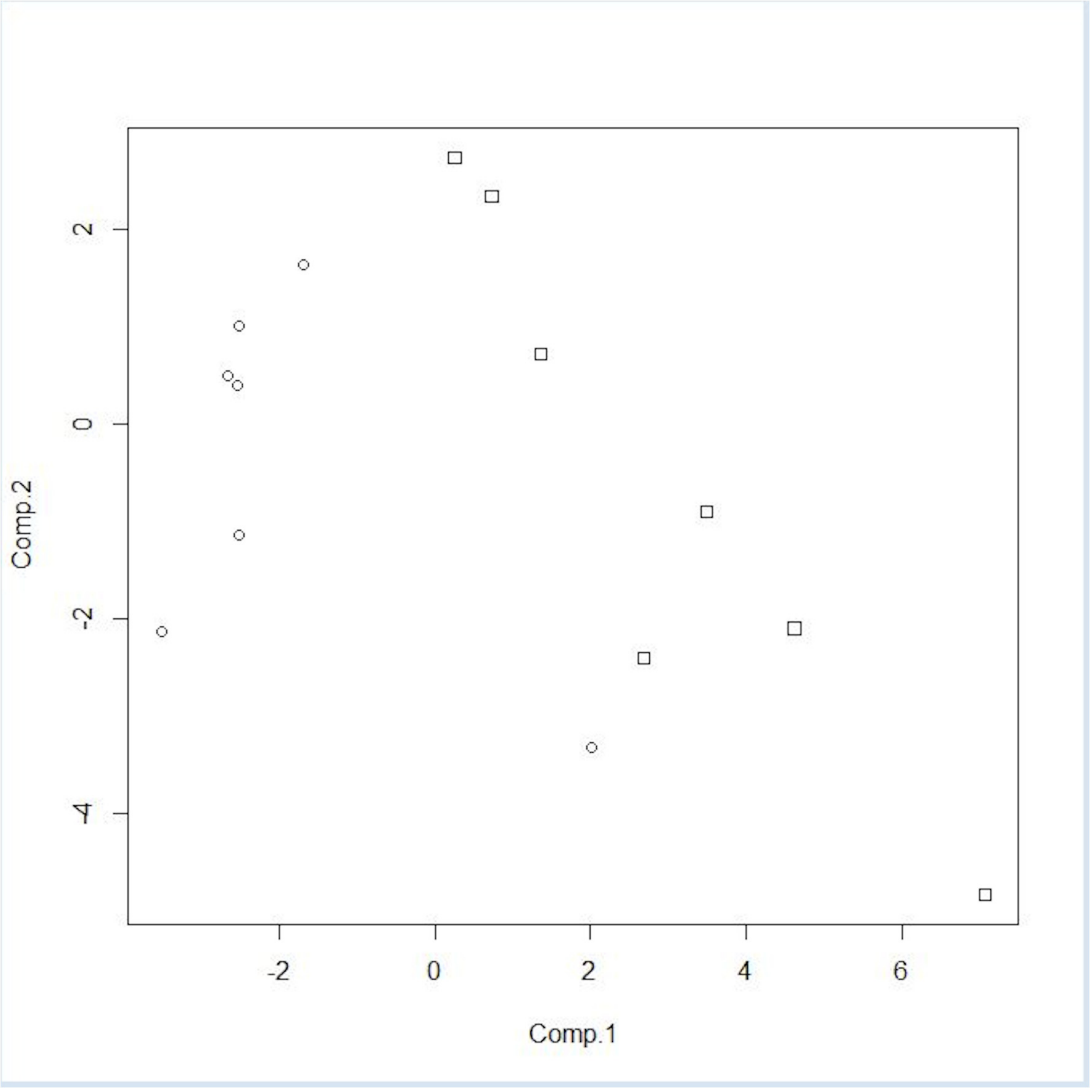
PCA score plot for non-vaccinated male and female WTD. Squares represent pre-challenge samples, circles represent post-challenge samples at 5 months. doi: http://dx.doi.org/10.6084/m9.figshare.1418311.

The classification models were developed from the ion intensity data acquired from the XCMS Online data analysis and transformed as PCA scores. Scatter plots (Figs 5–8) of the samples for the PCA transformed data using the first two principal component scores visually convey the separation of pre- and post-challenge samples across each pairwise comparison.

Principal components analysis scores were calculated for seven or eight ions in the vaccinated or non-vaccinated male WTD treatment groups when comparing pre- *versus* post-challenge chromatograms. For the non-vaccinated female treatment group eleven ions were used to calculate PCA scores. In the mixed-gender analysis of non-vaccinated WTD, eleven ions were used in the analysis as the median centering and variance scaling data transformation produced values of infinity for some data points for some samples.

The LDA classification model results for each of the four treatment comparisons based on two PCA scores (Figs 5–8) are summarized in Table 3. For both the non-vaccinated gender based analysis there were no misclassifications across the two sample sets using a model based on two PCA scores. The classification results for the analytical results incorporated into two PCA score models correspond to a test sensitivity and specificity each equal to 100%. The classification of samples in the mixed gender non-vaccinated WTD analysis had a total misclassification rate of 4.25% with a false negative rate of 0.5% and a false positive rate of 3.75%. The corresponding sensitivity was 94% with a specificity of 99%.

**Table 3:** Linear Discriminant Analysis classification model results for models based on 2 Principal Components Analysis scores derived from ion intensities for within group comparison pre- and post- *M*. *bovis* challenge.

	Vaccination Status			
	Non-vaccinated	BCG-Vaccinated	Non-vaccinated	Non-vaccinated
	Sex			
	male (n = 4)	male (n = 4)	female (n = 3)	mixed (n = 7)
Number of ions used in PCA	8	7	11	11
2 components				
Total miss classified	0%	0.67%	0%	4.25%
False negative	0%	0.33%	0%	0.5%
False Positive	0%	0.33%	0%	3.75%
Sensitivity	100%	99%	100%	94%
Specificity	100%	99%	100%	99%

The overall misclassification rate for the BCG-vaccinated male WTD treatment group across the two sample sets using a model based on two PCA scores was less than one percent, with the distribution being equally false positive and false negative classifications. The classification results for the analytical results incorporated into two PCA score models correspond to test sensitivity and specificity each equal to 99%.

**Fig 9:**
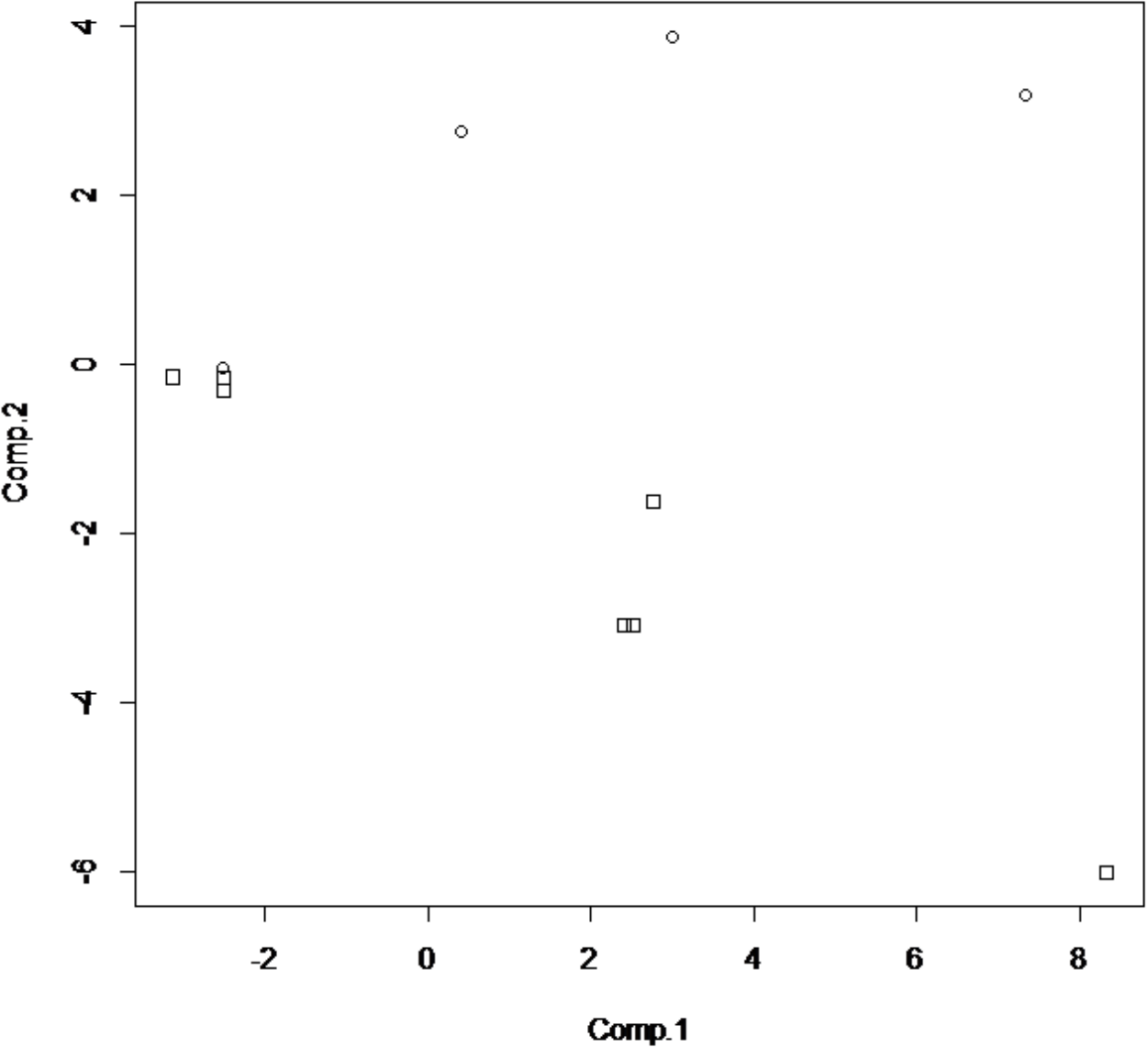
PCA score plot for the non-vaccinated male, non-vaccinated female, and vaccinated male WTD samples prior to *M*. *bovis* challenge. Squares represent non-vaccinated males or females, circles represent the vaccinated males. doi: http://dx.doi.org/10.6084/m9.figshare.1418309.

The ability to separate the non-vaccinated males and females from the vaccinated males, in a between group comparison, prior to challenge, based on a PCA score transformation of the peak areas for the compounds listed in Table 2 is apparent in Fig 9. The four vaccinated males lie in the upper half of the PCA space while the non-vaccinated males and females appear in the lower half. The LDA classification model based on 2 PCA scores provided a sensitivity of 81.8% and a specificity of 98.5% (Table 4). Models based on 3 or 4 scores had lower sensitivity and specificity.

**Table 4:** Linear Discriminant Analysis model results based on 2 to 4 PCA components derived from peak areas for predicting BCG-vaccination status pre-*M*. *bovis* challenge in a between group comaprison.

Number of PCA Components	2	3	4
Total miss classified	9.3%	13.3%	25.4%
False negative	8.3%	9.0%	10.7%
False Positive	1.0%	4.3%	14.7%
Sensitivity	81.8%	79.2%	77.8%
Specificity	98.4%	93.8%	81.0%

**Fig 10:**
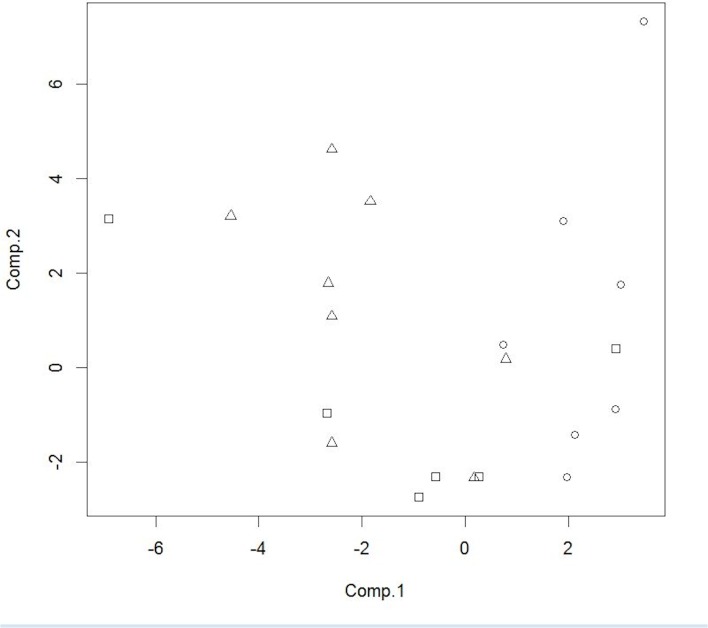
PCA score plot for three class WTD fecal sample cluster analysis. On the X-axis are the 1^st^ component scores, the on the Y-axis are the 2nd component scores. Squares represent pre-challenge samples from non-vaccinated WTD, circles represent post-challenge samples at 5 months from non-vaccinated WTD and triangles represent post-challenge samples at 5 months from vaccinated WTD. doi: http://dx.doi.org/10.6084/m9.figshare.1418315.

The PCA score plot for the three class model based on peak areas for the compounds listed in Table 2 is presented in Fig 10. The pre-challenge samples from the non-vaccinated WTD group are located at the lower left and lower center of the plot, while the post-challenge samples from the non-vaccinated WTD are located on the right side of the plot. The samples from the BCG-vaccinated WTD are grouped across the center of the plot with some overlap with the other two groups.

The classification results of the LDA model based on three PCA scores summed across the 100 iterations of model are presented in Table 5. This model provided the best representation of the data. One pre-challenge sample from a non-vaccinated female WTD was removed from the analysis as an outlier. The sensitivity and specificity for this model were 78.6% and 91.3%, respectively (Table 5). An example of the calculation of these values from model output can be found in the supporting information section (S1 Appendix).

**Table 5:** Sensitivity and specificity for the classification of fecal samples, based on three Principle Component Analysis scores, derived from peak areas, post-*M*. *bovis* challenge, in a mixed gender WTD population.

		Comparison		
		Samples from non-vaccinated deer pre-challenge	Samples from non-vaccinated deer post-challenge	Samples from vaccinated deer post-challenge
Total misclassified		16.3%	7.3%	20.3%
Miss classified as:				
	Pre Tb exposure	—	4.7%	17.3%
	Post Tb exposure	2.7%	—	3.0%
	Vaccinated	13.7%	2.7%	—
	Vaccinated	13.7%	2.7%	—
Sensitivity	78.6%			
Specificity	91.3%			

Details for the model used to classify a sample are presented in the text.

## Discussion

In this pilot study it was possible to discriminate between non-vaccinated WTD before and five months post-challenge with *M*. *bovis* strain 95–1315 based on our GC/MS analysis of a subset of VOCs found in fecal samples. Additionally, we were able to discriminate between BCG-vaccinated and non-vaccinated WTD, three months post vaccination. To the authors’ knowledge, this is the first report of a study examining VOCs in WTD, using feces as the target sample to identify animals with bovine tuberculosis.

Previous studies have demonstrated that analysis of breath VOCs can be performed to identify *M*. *bovis* infection of cattle or *M*. *tuberculosis* infection of humans [29–31]. Fundamental to these approaches is the application of a statistical process to identify the compounds that are significantly different across the treatment groups to allow for the development of a model. Our approach focuses on using single ions identified as significantly different across treatment groups in the MS chromatogram as a basis for identifying compounds to evaluate as biomarkers of infection. We used all the compounds (Table 2) identified in the pairwise comparisons across select subpopulations to evaluate the feasibility of using this larger group of compounds to distinguish between *M*. *bovis*-challenged WTD in a population where some individuals had been vaccinated. Further development of this approach may culminate in an efficient and inexpensive tool that not only effectively identifies animals with tuberculosis, but also can discriminate between vaccinated and non-vaccinated animals both before and after *M*. *bovis* exposure. However, the within-group effect of vaccination pre-*M*. *bovis* challenge on fecal VOC profiles could not be determined in this study due to the opportunistic nature of the samples collected during an ongoing vaccine trial [26].

Studies on the VOC profiles of ruminants have commonly focused on components identified as odorants, particularly volatile fatty acids, phenols, indole compounds, and amines originating from feed lots [32]. Many of the common volatiles observed reflect the composition of the diet, for example indole fluctuates in response to changes of high moisture versus dry-rolled corn in feed mix [33–35] and fenchol is commonly synthesized by plants [36]. The animals in this study all shared a common dietary history over the course of the study, and the food source was pelletized and homogeneous in composition. Many of the peaks in the chromatograms eliminated from consideration in this analysis after identification using a NIST library search are likely associated with metabolic products resulting from components in the diet. One compound, 4-methyl-phenol, has been identified as an important volatile cue in feces signaling reproductive status in water buffalo (*Bubalus bubalis*) females [37] and is not associated with diet.

The majority of the compounds in Table 2 are alcohols or ketones. Alcohols may result from cytochrome P450 hydrolysis of fatty acid peroxidation products [37] following lipid peroxidation at an unsaturated double bond in an alkyl chain. The compound 1-decanol has been observed in head space analysis over gram negative enteric bacteria including *E*. *coli*, and select members from the genera *Salmonella*, *Klebsiella and Enterobacter* [38, 39]. Lipases release methyl ketones from fatty acid alkyl chains and 2-nonanone is commonly produced from oleic acid [40, 41]. Additionally, the reduction of ketones may result in the formation of secondary alcohols. The effect of vaccination coupled with challenge to *M*. *bovis* strain 95–1315 changed the concentration of six compounds in male WTD, of which only three; 2-(1,1-dimethoxy)-ethanol, acetophenone, and 2-decanone were unique to the vaccinated WTD (Table 2). We were initially uncertain how fecal VOC profiles present in the BCG-vaccinated WTD might differ from those found in post-exposure fecal samples collected from non-vaccinated animals with evidence of *M*. *bovis* infection given that *M*. *bovis* BCG Danish is an attenuated form of *M*. *bovis*. However, the use of these six compounds in the classification of samples across three classes rarely resulted in misclassification of a BCG-vaccinated WTD as non-vaccinated animal with evidence of *M*. *bovis* infection post-challenge (Table 4). Thus classification appears to not require exclusively unique compounds but reflects the change in concentrations of compounds common across the groups.

The 17 compounds identified in the within-group comparisons provide a basis for distinguishing fecal samples collected from BCG-vaccinated WTD from those samples collected from non-vaccinated WTD (Table 5). The misclassifications observed in the LDAs were attributable to pre-challenge non-vaccinated WTD samples being classified as originating from BCG-vaccinated WTD, or BCG-vaccinated WTD samples being classified as originating from non-vaccinated pre-challenge WTD. The inferences that can be drawn from this study are constrained by the low numbers of samples that were available and the inability to collect fecal samples from a cohort of non-vaccinated non-challenged deer over the entire course of the study.

It would be difficult to make inferences as to the role of vaccination or exposure to *M*. *bovis* on the physiological processes that might account for the patterns observed in the chromatograms acquired from the fecal head space samples. Breath samples from tuberculosis infected humans or other mammals often contain compounds that can be directly associated with mycobacterial metabolism [29, 42]. A review of the literature indicates that *M*. *bovis* is rarely present in the feces of WTD whether samples were obtained from experimentally or naturally infected WTD [14, 43]. No gastrointestinal lesions were noted in any of the deer involved in this study. Thus, the VOC profiles acquired from the fecal samples analyzed are likely not directly attributable to the presence of *M*. *bovis* in the gastrointestinal tract. The diversity of gut microbiota has been shown to decrease in a mouse model following infection with *M*. *tuberculosis* [44]. These changes in diversity were observed to occur rapidly after infection and result in a different population distribution, particularly in the classes *Clostridiales* and *Bacteriodales*, following infection. Species from these two classes of microbiota have been demonstrated to have significant roles in lipid metabolism in ruminants [45, 46]. Anticipating that similar changes in gut microbiota occur in WTD, we would suggest that these might account for the changes observed in the VOC profiles as many of the compounds identified in our study are associated with lipid metabolism.

The PCA classification models developed from the identified ion intensity data allowed for the discrimination of samples collected from animals pre- and post-*M*. *bovis* strain 95–1315 challenge. The PCA score plots visually convey the separation of treatment groups in pair-wise between-group and among-group comparisons. Only a small number of features, or compound peak areas, identified in the chromatograms are required to make the distinction in these models. This is consistent with the work of Scott-Thomas et al. [42] which demonstrated the use of 4 compounds as markers for a breath test to identify *M*. *tuberculosis* infection in human subjects.

The unique result from this pilot study is the ability to tentatively classify fecal samples as originating from BCG-vaccinated or non-vaccinated WTD pre-and post- *M*. *bovis* challenge. Future work should address the feasibility of monitoring populations of WTD in areas where endemic *M*. *bovis* infection is present. The ability to distinguish BCG-vaccinated from *M*. *bovis*-infected WTD may provide an important tool with which to measure BCG vaccine uptake and efficacy, should BCG vaccination be implemented in the Michigan WTD population where *M*. *bovis* is endemic. In addition, these methods may be applied to other domestic and wildlife species in order to detect and control bovine tuberculosis.

## Supporting Information

S1 Appendix An example of the calculation for Sensitivity (Sn) and (Sp) for the three class model. (DOCX)
