# Supplementary material for: Fecal Volatile Organic Ccompound Profiles from White-Tailed Deer (Odocoileus virginianus) as Indicators of Mycobacterium bovis Exposure or Mycobacterium bovis Bacille Calmette-Guerin (BCG) Vaccination
Source: PLoS One. 2015 Jun 10;10(6):e0129740. doi: 10.1371/journal.pone.0129740 (PMC4465024; doi:10.1371/journal.pone.0129740)
Supplement: S1 Appendix — (DOCX) [file pone.0129740.s001.docx]

**Supplemental Information**

**S1 Appendix. An example of the calculation for Sensitivity (Sn) and (Sp) for the three class model.**

| **Classification data used to determine diagnostic sensitivity and specificity.** | | | |
| --- | --- | --- | --- |
|  | Non-vaccinated WTD | Non-vaccinated WTD | BCG vaccinated WTD |
|  | Negative | Infected | Negative |
| Correctly classified: | 27 | 81 | 60 |
| Classed as infected: | 8 | 14 | 52 |
| Classified as BCG vaccinated: | 41 | 8 | 9 |

| **Sum of correct and misclassified samples across models.** | | | | |
| --- | --- | --- | --- | --- |
| True positive | True negative | False positive | False negative |  |
| 81 | 27 | 8 | 14 |  |
|  | 41 | 9 | 8 |  |
|  | 60 |  |  |  |
|  | 52 |  |  |  |
| 81 | 180 | 17 | 12 | Total |

| **Notation for Calculating Sensitivity and Specificity.** | | | |
| --- | --- | --- | --- |
|  | Lesions/infection noted | No lesions/infection noted |  |
| Test positive: | 81 | 17 |  |
| Test negative: | 22 | 180 |  |
|  | 103 | 197 | Total |
|  |  |  |  |
| Sensitivity: | 71/ 103 = 78.6% |  |  |
| Specificity: | 180/ 197 = 91.3% |  |  |
